# RNA m^5^C regulator-mediated modification patterns and the cross-talk between tumor microenvironment infiltration in gastric cancer

**DOI:** 10.3389/fimmu.2022.905057

**Published:** 2022-10-27

**Authors:** Qiang Zhang, Xiangfei Sun, Jianyi Sun, Jiangshen Lu, Xiaodong Gao, Kuntang Shen, Xinyu Qin

**Affiliations:** ^1^ Department of General Surgery, Zhongshan Hospital, Fudan University, Shanghai, China; ^2^ Department of Gastrointestinal Surgery, the Second People’s Hospital of Lianyungang Affiliated to Kangda College, Nanjing Medical University, Lianyungang, Jiangsu, China

**Keywords:** 5-methylcytosine (m^5^C), RNA methylation modification, tumor microenvironment, immune, prognosis, m^5^Cscore

## Abstract

The effect of immunotherapy strategy has been affirmed in the treatment of various tumors. Nevertheless, the latent role of RNA 5-methylcytosine (m^5^C) modification in gastric cancer (GC) tumor microenvironment (TME) cell infiltration is still unclear. We systematically explore the m^5^C modification patterns of 2,122 GC patients from GEO and TCGA databases by 16 m^5^C regulators and related these patterns to TME characteristics. LASSO Cox regression was employed to construct the m^5^Cscore based on the expression of regulators and DEGs, which was used to evaluate the prognosis. All the GC patients were divided into three m^5^C modification clusters with distinct gene expression characteristics and TME patterns. GSVA, ssGSEA, and TME cell infiltration analysis showed that m^5^C clusters A, B, and C were classified as immune-desert, immune-inflamed, and immune-excluded phenotype, respectively. The m^5^Cscore system based on the expression of eight genes could effectively predict the prognosis of individual GC patients, with AUC 0.766. Patients with a lower m^5^Cscore were characterized by the activation of immunity and experienced significantly longer PFS and OS. Our study demonstrated the non-negligible role of m^5^C modification in the development of TME complexity and inhomogeneity. Assessing the m^5^C modification pattern for individual GC patients will help recognize the infiltration characterization and guide more effective immunotherapy treatment.

## Introduction

As a global disease, gastric cancer (GC) is the fifth most diagnosed malignancy and the third most common cause of cancer-related death, with 784,000 deaths worldwide in 2018 (1). Although the incidence and mortality rates of GC have declined in several countries, regions seriously threatened by GC, such as China and other East Asian countries, still bear severe health and economic burden. In China, 562,000 newly diagnosed GC patients were recorded, accounting for nearly half of the new cases worldwide (2). The 5-year survival rate of GC is 35.9% in China due to the late stage at diagnosis, notably lower than 71.5% in South Korea and 65% in Japan (3, 4). Due to the complexity of the pathogenic mechanism and the lack of specific biomarkers of GC, the effects of treatment strategies such as surgery, chemotherapy, and radiotherapy are not satisfactory.

RNA 5-methylcytosine (m^5^C) is an important kind of RNA methylation modification; there have been more than 150 RNA modifications identified to date (5). Traditionally, DNA m^5^C has been proven to be the most dominant DNA modification in mammals and functions by adding a methyl group at the carbon-5 position of the cytosine base (6). RNA m^5^C modification, as the third layer of epigenetics, can be found in but not limited to mRNA, noncoding RNA, and tRNA (7–13). Like other RNA epigenetic modifications, such as N6-methyladenosine (m^6^A), m^5^C is a dynamic reversible process that can be regulated by “writers”, “erasers”, and “readers”, namely, the methyltransferases, demethylases, and binding proteins (14). The methylation formation of m^5^C modification is catalyzed by methyltransferases composed of the SUN/NOL1/NOP2 domain family of proteins (NSUN1, NSUN2, NSUN3, NSUN4, NSUN5, NSUN6, and NSUN7) and DNA methyltransferase homologues (DNMT1, DNMT2, DNMT3A, and DNMT3B) (15–17). At the same time, the demethylation process is regulated by the erasers consisting of enzymes of the ten-eleven translocation (TET) family members, including TET1, TET2, and TET3 (18, 19). Additionally, the whole methylation process is mediated by a cluster of special RNA-binding proteins, including ALYREF and YBX1 (8, 20). An increasing number of studies validate that the dynamic modification of m^5^C and its regulators is involved in a series of physiological and pathological processes, including RNA stability, gene expression, and protein synthesis. As for tumor malignant biological behaviors, it has been reported that m^5^C and its regulators play essential roles in the pathogenesis of leukemia (21), hepatocellular carcinoma (HCC) (22), glioblastoma multiforme (GBM) (12), and bladder cancer (20), indicating the promising prospect of m^5^C modification in cancer treatment.

Recently, immunotherapy, anti-PD-L1 antibody, and anti-PD-1 antibody have increased the overall survival rate of some advanced GC patients who were treated with two or more lines of chemotherapy (23). The efficiency of immunotherapy depends on the status of EB virus infection, microsatellite instability (MSI)/mismatch repair (MMR), and the expression of PD-L1. However, the dominant population of immunotherapy is still challenging to identify because of the heterogeneity of GC. Hence, to better analyze the heterogeneity and immunophenotype of patients with GC, it is essential to improve long-term survival. Consistently, epigenetic and genetic variations of malignant cells are the only factors participating in the tumor progression, which is a complex multistep process. Notwithstanding, numerous studies have proved that the tumor microenvironment (TME), where tumor cells survive and grow, is crucial in tumorigenesis and development. The composition of TME is rather complicated, including not only the tumor part but also the stromal cells, macrophages, bone marrow-derived cells (BMDCs), distant recruited cells, secreted factors, and neovascularization (24). The detailed types of cells and cytokines in the TME are complex, including cancer-associated fibroblasts (CAFs), myeloid cells, lymphocytes, chemokines, cytokines, and growth factors. Among these cells, tumor-associated macrophages (TAMs), tumor-associated neutrophils (TANs), myeloid-derived suppressor cells (MDSCs), Tie2-expressing monocytes, and dendritic cells together constitute the tumor-associated myeloid cells (TAMCs) (25). The cross-talk between cancer cells and TME components promotes tumor proliferation and angiogenesis, avoids hypoxia, inhibits apoptosis, and mediates immune tolerance. With the gradual deepening of the understanding of the complexity and diversity of TME, increasing data depict its essential role in immune escape and immunotherapy. Moreover, the TME cell infiltration pattern can predict the response to the immune checkpoint blockade (ICB), which is promising in the tumor treatment strategies (26). Accordingly, particular tumor immunophenotypes are supposed to be validated *via* thoroughly parsing the TME landscape complexity and heterogeneity (27). As GC is characterized by tumor heterogeneity, it is urgent to identify the dominant population of immunotherapy by the landscape TME cell infiltration.

Lately, m^5^C modification is related to the TME-infiltrating immune cells, and the mechanisms are more complicated than expected. In systemic lupus erythematosus (SLE), abnormal m^5^C mRNAs were identified as relevant to critical immune pathways in CD4+ T cells (28). Another study reported that the eraser TET1 is downregulated *via* NF-κB signaling pathway activation in breast cancer cells (29). Interestingly, Andries and colleagues found that m^5^C-modified mRNA promoted protein expression by the increased ability of the mRNA to elude downstream innate immune signaling and activation of endosomal Toll-like receptor 3 (TLR3) (30). During virus infection, m^5^C RNA methyltransferases, such as NSUN family proteins, were employed to modify viral RNA and change antiviral host responses (31). All these latest findings reveal the fact that m5C modification and regulators may have a further effect on the TME, and previous studies focus only on one or two m^5^C regulators due to the limitation of technologies.

In the present study, the genomic and clinical data of 1,983 GC samples were employed to thoroughly estimate the m^5^C modification patterns and the correlation between m^5^C modification and TME features. Three different m^5^C modification patterns and the specific TME cell infiltration peculiarities were identified. Three immunophenotypes, immune-inflamed, immune-excluded, and immune-desert phenotype, were related to the three m^5^C clusters. Subsequently, a scoring system based on the m^5^C modification pattern was established for individual GC patients.

## Materials and methods

The detailed materials and methods can be found in the **supplementary files** (32–37).

## Results

### Blueprint of genetic variation of m^5^C regulators in GC

**Figure 1:**
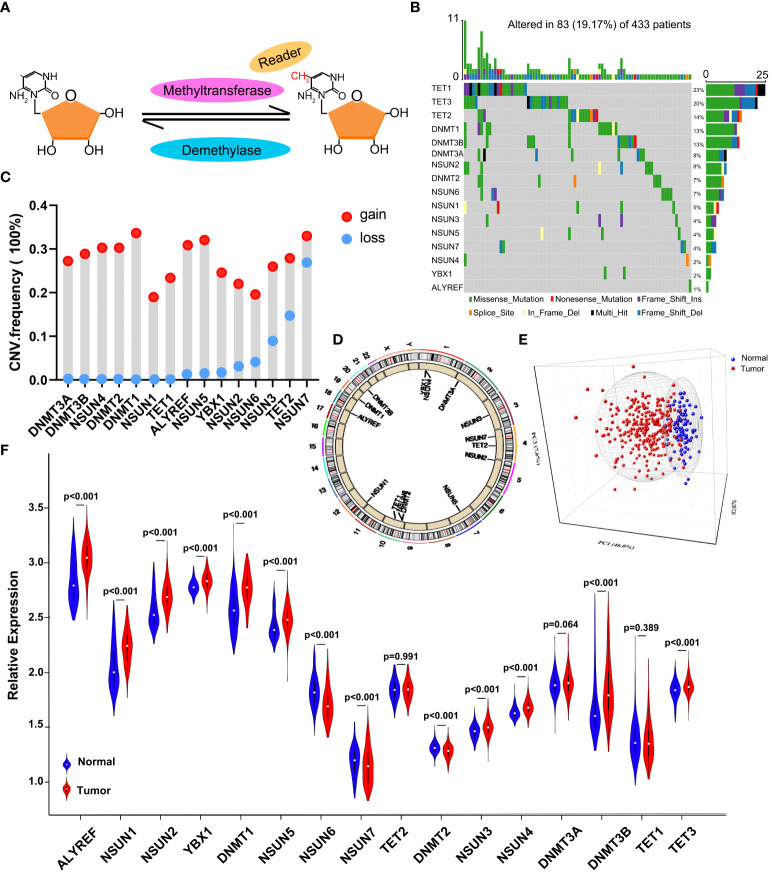
The landscape of genetic and expression alternation of m^5^C regulators in GC. **(A)** Diagram of the dynamic reversible process of m^5^C RNA methylation regulated by different types of regulators. **(B)** The mutation frequency of 16 m^5^C regulators in 433 GC patients from the TCGA-STAD cohort. Each column represents individual patients. The upper bar plot indicates TMB. The numbers on the right show the mutation frequency of specific regulators and the right bar plot represents the proportion of each variant type. The colors of each variant type are listed at the bottom. **(C)** The CNV mutation frequency of m^5^C regulators in the GSE62717 dataset. The height of the column represents the variation frequency. Amplification frequency, red dot; deletion frequency, blue dot. **(D)** The location of CNV mutation of 16 m^5^C regulators on 23 chromosomes in the GSE62717 dataset. **(E)** PCA indicates that the expression of 16 m^5^C regulators can distinguish GC tumors from normal samples in the GSE2269 cohort. Tumors and normal samples are marked with blue and red, separately. **(F)** The differential expression of 16 m^5^C regulators between normal and tumor samples. Tumor, red; normal, blue.

In the process of dynamic modification, methyltransferases and demethylases work together to keep the balance of the RNA m^5^C modification with the help of the readers. The ideograph of RNA m^5^C modification is shown in **Figure 1A**. Firstly, the characteristics of somatic mutations and copy number variations (CNVs) of the 16 m^5^C regulators were summarized in GC. Among all the 433 samples from TCGA, 83 (19.17%) patients experienced mutations of m^5^C regulators. We found that the three demethylases exhibited the highest mutation rates, while the readers (YBX1 and ALYREF) hardly showed any mutations (**Figure 1B**). Moreover, a significant mutation co-occurrence pattern was identified between NSUN2 and NSUN3 (**Figure S1B**). For CNV analysis, the most prevalent CNV alternation in the regulators was the amplification in copy number, except for NSUN3, TET2, and NSUN7, which were characterized by a high frequency of CNV deletion (**Figure 1C**). In **Figure 1D**, the detailed locations of CNV alternation of each m^5^C regulator are recorded on the chromosomes. Notably, we could thoroughly determine GC patients from normal samples based on the expression of the 16 m^5^C regulators (**Figure 1E**). To further ascertain the relation between the above genetic alternations and the expression of m^5^C regulators, we explored the expression of regulators in both GC and normal tissues. We found that CNVs might be the main factors leading to the abnormal expression of the m^5^C regulators. Regulators with amplificated CNV tended to highly expressed in tumor samples (e.g., DNMT1, ALYREF, and NSUN5), and *vice versa* (e.g., NSUN7 and NSUN6) (**Figures 1C, F**). The assessment disclosed the heterogeneity of expressional and genetic alternation patterns in m^5^C regulators between GC and normal tissues, hinting that the aberrant expression of m^5^C regulators played an essential role in the tumorigenesis and development of GC.

### m^5^C methylation modification patterns mediated by 16 regulators

**Figure 2:**
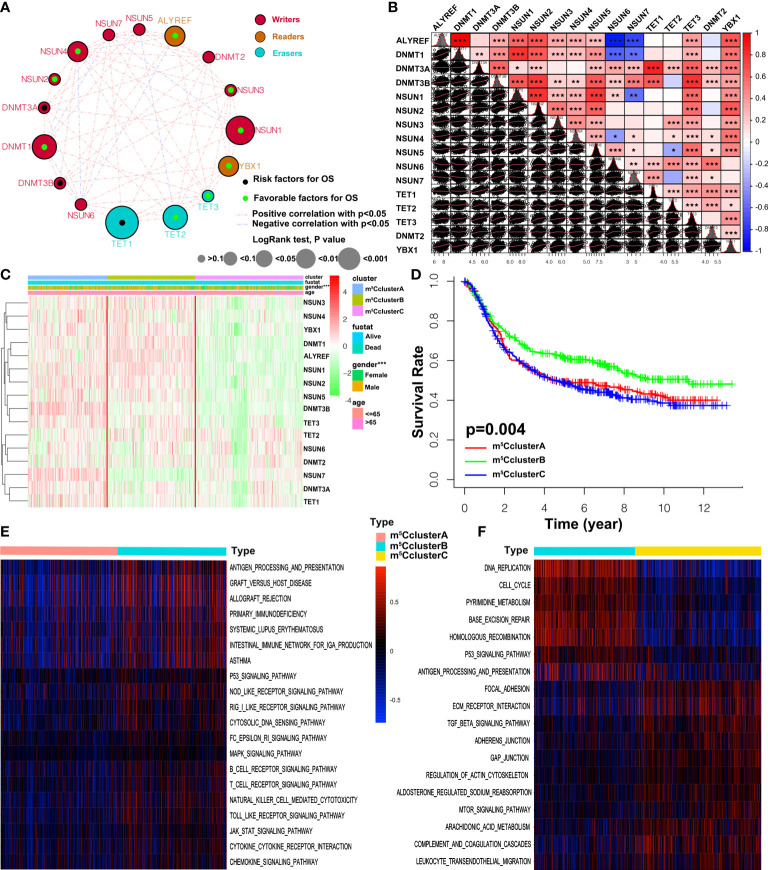
m^5^C methylation modification patterns and biological characteristics. **(A)** The interaction between 16 m^5^C regulators in GC. The size of the circles represents the effect of a specific regulator on the prognosis, and the values calculated *via* the Log-rank method were *p* > 0.1, *p* < 0.1, *p* < 0.05, *p* < 0.01, and *p* < 0.001, respectively. Black dots, favorable factors; green dots, risk factors. The lines between two regulators show the interaction. Negative correlation and positive correlation are marked with blue and red. **(B)** The expression correlation of the 16 m^5^C regulators in GC. Each cell in the lower left corner represents the expression of two regulators, and the red line is the trend line. Cells in the upper right corner show the statistic result. The asterisks represent the *p*-value (*p < 0.05, **p < 0.01, and ***p < 0.001). **(C)** Unsupervised clustering of 16 m^5^C regulators in the five independent gastric cancer cohorts. The m^6^A clusters and basic clinical information are used as patient annotations. Each column represents patients and each row represents m^5^C regulators. **(D)** Overall survival of three m^5^C modification clusters based on the GC patients from five GEO cohorts. Log-rank *p*-value 0.004 indicates a significant prognostic difference among three m^5^C clusters. **(E, F)** GSVA enrichment analysis revealing the activation states of biological pathways in distinct m^5^C clusters. The heatmap is employed to visualize the biological processes, and blue represents inhibited pathways and yellow represents activated pathways.

A meta-cohort including five GEO datasets (GSE57303, GSE84437, GSE34942, GSE62254, and GSE15459) with full OS and other clinical data was used to identify the expression pattern of 16 regulators. The prognostic values of 16 m^5^C regulators were analyzed through a univariate Cox regression model (**Figure S1C** and **Figure 2A**). We found that the readers ALYREF and YBX1 were favorable prognosis factors for GC patients. The cross-talk between 16 regulators and prognostic significance for patients was revealed in the m^5^C regulator network (**Figures 2A, B**). We noticed that a significant correlation was shown in both the same and different functional category regulators. Interestingly, the correlation of expression is consistent in regulators from the same functional category. However, we found that the relationship in writers is much complicated, such as DNMT1, which is remarkably negatively correlated with NSUN6 and NSUN7 (**Figure 2B**). In addition, the expression of the readers ALYREF and YBX1 was almost significantly correlated with other regulators. According to the expression of 16 m^5^C regulators, we further explored the m^5^C modification patterns *via* the ConsensusClusterPlus package, and identified three different modification patterns by the unsupervised clustering method, including 308 patients in m^5^C cluster A, 334 patients in m^5^C cluster B, and 417 patients in m^5^C cluster C (**Figures S2A–D** and **Table S3**). The heatmap of the 16 m^5^C regulators in 1,059 GC patients is depicted in **Figure 2C**. The expression of 16 regulators in three m^5^C clusters was remarkably different. LogRank analysis showed that the prognosis of patients in m^5^C cluster B was better than the other two clusters (**Figure 2D**).

### TME cell infiltration characteristics in distinct m^5^C modification patterns

**Figure 3:**
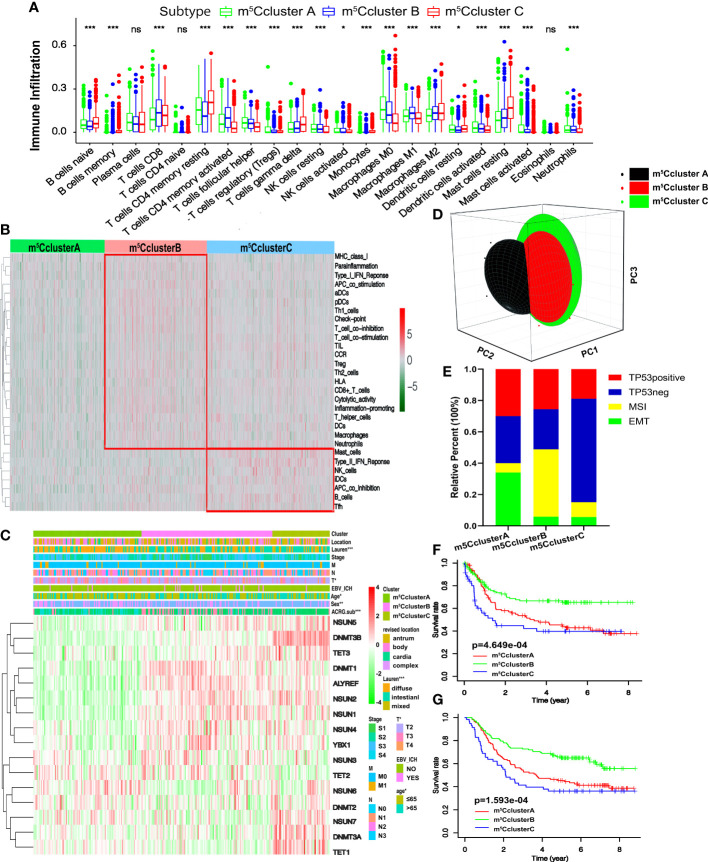
TME cell infiltration traits and transcriptome characteristics in three m^5^C modification clusters. **(A)** The enrichment of TME-infiltrating cell in distinct m^5^C clusters. The lower and upper ends of the boxes represent interquartile range of values. The dots represent outliers and the lines in the boxes show the median value. **(B)** The heatmap of TME-infiltrating cells in three m^5^C patterns. **(C)** Unsupervised clustering of 16 m^5^C regulators in the ACRG cohort. The m^5^C cluster, location, Lauren types, tumor stage, EBV status, sex, age, and ACRG molecular subtypes are used as patient annotations. High and low expression of regulators is marked with red and green, respectively. **(D)** PCA shows that the expression of 16 m^5^C regulators can divide the ACRG cohort into three distinct m^5^C clusters. **(E)** The relative percent of four molecular subtypes in distinct m^5^C clusters. **(F)** Recurrence-free survival of three m^5^C modification clusters based on the GC patients from the ACRG cohort. **(G)** Overall survival of three m^5^C modification clusters based on the GC patients from the ACRG cohort (ns, not significant: *p < 0.05; **p < 0.01; ***p < 0.001).

To better understand the biological characteristics among the distinct m^5^C modification clusters, the GSVA enrichment method was conducted. In **Figure 2E**, m^5^C cluster A is related to the immune suppression process, while m^5^C cluster B is notably enriched in immune full activation pathways, including cytokine–cytokine receptor interaction, natural killer cell-mediated cytotoxicity, antigen processing and presentation, Toll-like receptor signaling pathway, and chemokine signaling pathway. m^5^C cluster C is enriched in carcinogenic and stromal activation pathways, such as ECM receptor interaction, TGF beta signaling pathway, adhesion and gap junction, mTOR, and MAPK signaling pathways (**Figure 2F**). Interestingly, TME immune cell infiltration analysis subsequently showed that m^5^C cluster C was rich in resting and naïve immune cells, such as dendritic cells, CD4 memory T cells, mast cells, B cells, and other innate immune cells, by the CIBERSORT method. On the contrary, m^5^C cluster B is characterized by specific immune cell enrichment (**Figure 3A**, **Figure S2E**, and **Table S4**). The correlation of specific m^5^C regulators and immune cell is shown in **Figure S2F**. To further reveal the TME features, the single-sample GSEA (ssGSEA) analysis of all the 1,059 cases was conducted. In addition to immune cells, more details about immune functions and pathways can be summarized *via* the ssGSEA method. As shown in **Figure 3B**, three distinct immune patterns under three m^5^C clusters are identified (**Table S5**). Combined with the survival results above, we were surprised to find that m^5^C cluster A belonged to the immune-desert phenotype, characterized by immunological suppression; m^5^C cluster B was classified as immune-inflamed phenotype, which features immune activation and immune cell infiltration; m^5^C cluster C was labeled as immune-excluded phenotype, characterized by stromal activation and innate immune cell infiltration (**Figures 2D** and **3A, B**). These results demonstrated that the interaction among the writers, erasers, and readers might play fundamental roles in distinct m5C modification patterns and TME cell infiltration characteristics of individual GC patients.

### m^5^C methylation modification patterns in the ACRG cohort

We focused on the ACRG cohort, a group of 300 GC participants with complete clinicopathological information, to further reveal the biological behaviors and the features of m^5^C modification patterns. Like the meta-cohort datasets, the ACRG cohort is divided into three distinct m^5^C modification clusters as well by the unsupervised clustering method (**Figures S3A–D** and **Figures 3C, D**). The heatmap based on the expression of 16 m^5^C regulators shows that m^5^C cluster A exhibits a high expression of TET2 and NSUN6 and is downregulated in other regulators; m^5^C cluster B is characterized by the upregulated readers and five writers including NSUN1–4 and DNMT1; m^5^C cluster C shows high levels of two erasers and four writers (**Figure 3C**). We found that patients in m^5^C cluster A were rich in the diffuse subtype and tended to have poor differentiation. Intestinal subtype was more likely to be observed in m^5^C cluster B and C modification pattern. GC patients with diffuse histological and EMT molecular subtypes were related to poorer survival; on the contrary, MSI was linked to a better prognosis. Consequently, patients with an m^5^C cluster pattern were markedly linked to high malignancy, stromal activation, and poor prognosis (**Figure 3C**). Moreover, **Figure 3E** shows that patients in m^5^C cluster A are also significantly related to EMT molecular subtypes, while the MSI subtypes are featured by m^5^C cluster B modification. The survival results revealed that patients in m^5^C cluster B are related to a favorable prognosis, while m^5^C clusters A and C show a shorter survival time (**Figure 3F**). Notably, we also found that the relapse-free survival (RFS) of m^5^C cluster B is better than the other two clusters (**Figure 3G**). The findings above demonstrate that most GC patients with EMT molecular subtypes were divided into m^5^C cluster A and related to stromal activation; most patients with MSI instead of the EMT subtype were in m^5^C cluster B and characterized by immune activation.

### Immunomodulatory effect of m^5^C modification on the TME

**Figure 4:**
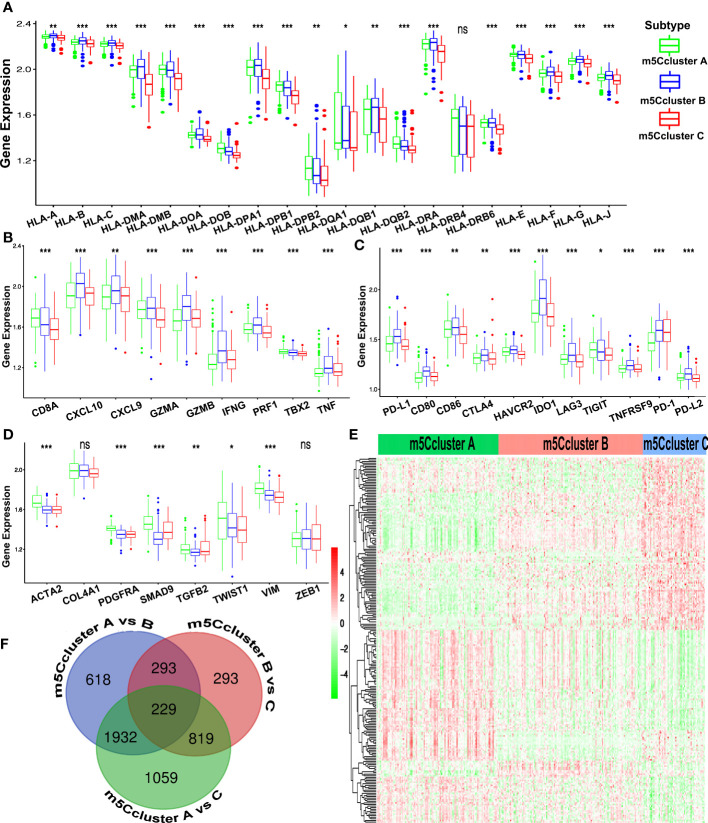
Immune characteristics of the different m^5^C patterns from the ACRG cohort. **(A)** The expression of the HLA alleles of GC patients of distinct m^5^C clusters. **(B)** Difference in the immune-activation-related gene expression among three m^5^C clusters. **(C)** Difference in the immune-checkpoint-related gene expression among three m^5^C clusters. **(D)** Difference in the TGF-β–EMT-related gene expression among three m^5^C clusters. **(E)** The Venn diagram shows the 229 DEGs from the pairwise comparison of three m^5^C clusters. **(F)** The heatmap of DEGs of the distinct m^5^C clusters reveals three expression patterns. (ns, not significant; *p < 0.05; **p < 0.01; ***p < 0.001).

Subsequently, four gene clusters belonging to distinct immune processes were used to reveal the role of m^5^C modification on the immune regulation of the TME. Chemokines and cytokines with different functions were selected from the published literature. The essential members of human leukocyte antigen (HLA), the major histocompatibility complex (MHC) of human beings, present antigen and mediate immune response. CD8A, CXCL9, CXCL10, GZMA, GZMB, IFNG, PRF1, TBX2, and TNF are related to immune activation. CD80, CD86, HAVCR2, CTLA-4, LAG3, IDO1, PD-1, PD-L1, PD-L2, TNFRSF9, and TIGIT are supposed to correlate with immune checkpoints. ACTA2, CLDN3, COL4A1, SMAD9, TGRB1, TGFBR2, TWIST1, VIM, and ZEB1 are considered to associate with TGF-β and EMT pathways (24, 38). In **Figure 4A**, HLA-I molecules, including HLA-A, B, C, E, F, and G, are remarkably highly expressed in m^5^C cluster B, which means stronger antigen presentation and tumor-killing ability. We noted that HLA-II molecules, such as HLA-DPB2, HLA-DQA1, HLA-DQB2, and HLA-DQA1, were upregulated in m^5^C cluster A. HLA-G is reported to suppress the immune response and leads to long-term immune escape and tolerance (39). Meanwhile, we also found that the expression of genes related to TGF-β and EMT pathways was remarkably upregulated in m^5^C cluster A, which added the evidence of stromal activation, while m^5^C cluster B exhibited overexpression of mRNAs relevant to immune activation (**Figures 4B–D**). Immune checkpoint analysis showed that all the genes, including CTLA-4, PD-1, and PD-L1, were upregulated in m^5^C cluster B (**Figure 4C**). The results above demonstrate that m^5^C modification patterns are significantly related to TME immune regulation and may play crucial roles in immunotherapy. However, these findings were only based on the 16 m^5^C modification regulators.

Considering the heterogeneity and complexity of m^5^C methylation modification, we tried to identify the DEGs under different m^5^C clusters using the limma package. Finally, 229 m^5^C phenotype-related DEGs were found and showed a distinct expression pattern under three m^5^C clusters (**Figures 4E, F**). The GO and KEGG enrichment analysis of the 229 DEGs showed that (**Figures S3E, F**) the DEGs were rich in immune-related biological processes and pathways, including CD8+ αβT cell activation, negative regulation of the immune system process, NOD-like receptor signaling pathway, and TNF signaling pathways.

### Generation of m^5^Cscore and capability to predict prognosis

**Figure 5:**
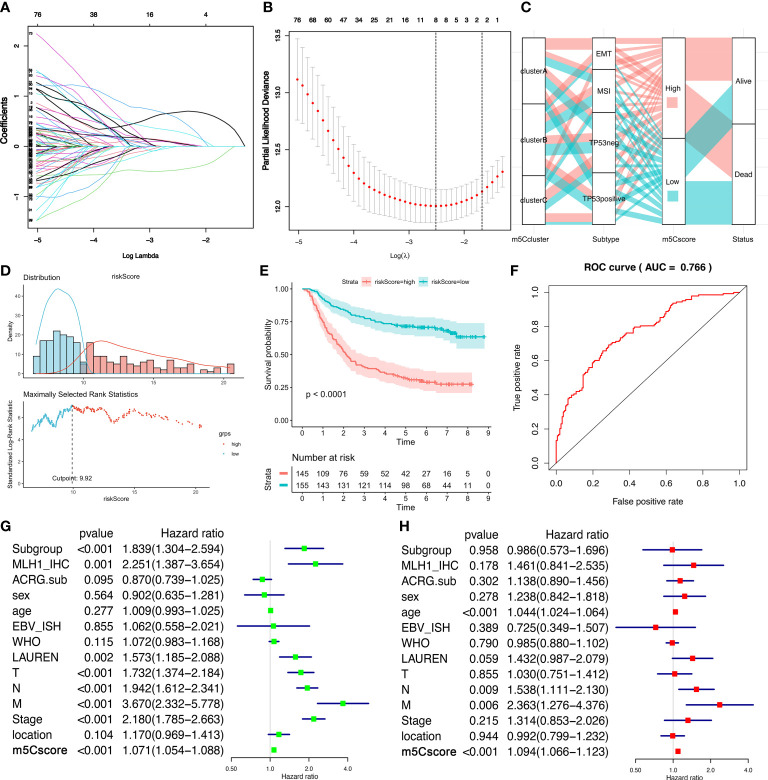
The generation of the m^5^Cscore system in the ACRG cohort. **(A, B)** The LASSO regression model and verification. **(A)** The longitudinal, lower transverse, and upper transverse coordinates are the correlation coefficient, Log Lambda (penalty coefficient), and the number of non-zero coefficients in the model. The lines with different colors show the trajectories of related variables changing with Log Lambda in the model. **(B)** The upper transverse, lower transverse, and longitudinal coordinates represent the format of the factor, the Log Lambda (penalty coefficient), and the error of cross-verification. The point with the smallest cross-verification error corresponds to the number of factors involved in the LASSO regression model. **(C)** Alluvial diagram demonstrates the changes of m^6^A clusters, molecular subtypes, m^5^Cscores, and status. **(D)** The distribution of the m^5^Cscore of each patient from the ACRG cohort. The cutoff point of m^5^Cscore is 9.92. **(E)** Kaplan–Meier curves show that the m^5^Cscore is significantly related to the overall survival of GC patients in the ACRG cohort, of which 145 patients were in the high-m^5^Cscore group and 155 patients were in the low-m^5^Cscore group (*p* < 0.0001, Log-rank test). **(F)** The predictive value of m^5^Cscore in the ACRG cohort. AUC, 0.766. **(G)** Univariate Cox regression analysis for m^5^Cscore in the ACRG cohort shown by the forest plot. **(H)** Multivariate Cox regression analysis for m^5^Cscore in the ACRG cohort shown by the forest plot.

**Figure 6:**
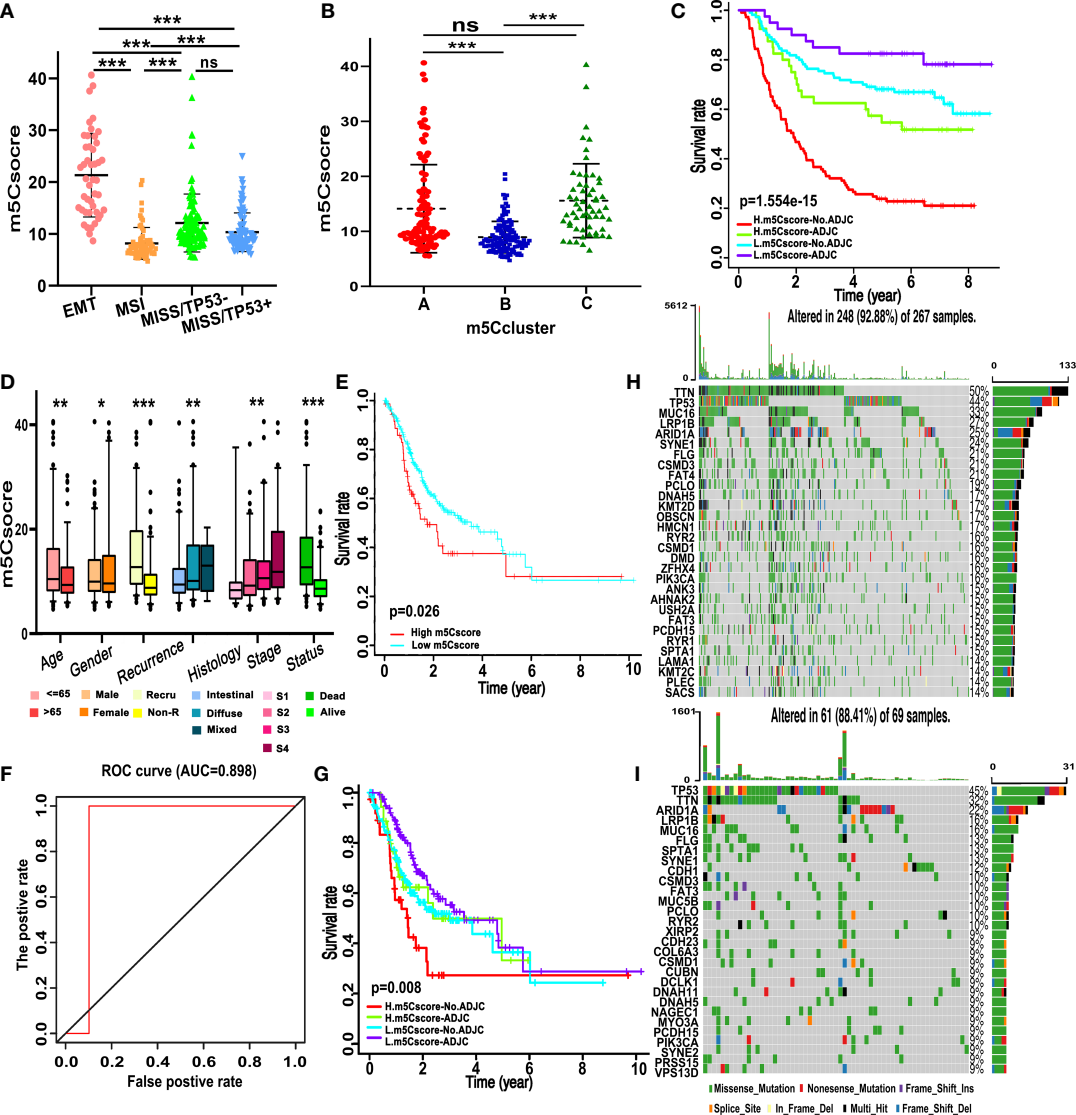
Traits of m^5^C modification in different molecular subgroups and somatic tumor mutation. **(A)** Differences in m^5^Cscore between different ACRG molecular subgroups. **(B)** Differences in m^5^Cscore between distinct m^5^C clusters. **(C)** Survival analyses for subgroup GC patients stratified by both adjuvant chemotherapy treatment and m^5^Cscore using the Kaplan–Meier method. L, low; H, high; ADJC, adjuvant chemotherapy. **(D)** Differences in m^5^Cscore among clinicopathological groups. **(E)** Kaplan–Meier curves show that the m^5^Cscore is significantly correlated to the overall survival of patients in the TCGA-STAD cohort. **(F)** The predictive value of m^5^Cscore in the TCGA-STAD cohort. AUC, 0.898. **(G)** Survival analyses for subgroup GC patients divided by adjuvant chemotherapy treatment and m^5^Cscore using Kaplan–Meier curves in the TCGA-STAD cohort. **(H,I)** The waterfall plot of tumor somatic mutation based on those with high m^5^Cscore **(H)** and low m^5^Cscore **(I)** in the TCGA-STAD cohort. Each column represents each GC patient. The upper and right bar plots show TMB and the proportion of each variant type. The number on the right indicated the mutation frequency in each gene. (ns, not significant; *p < 0.05; **p < 0.01; ***p < 0.001).

We established a scoring system that depended on the expression of DEGs and m^5^C regulators to quantify the individual m^5^C modification pattern; we termed this m^5^Cscore. The univariate Cox regression method was employed to determine the DEGs that were significantly related to the survival of GC patients in ACRG (**Table S6**). Ninety-nine genes were involved in the LASSO Cox regression algorithm to generate the m5Cscore signature, and eight genes were selected, including seven DEGs (RBPMS2, TNFRSF11A, NBEA, INHBB, RGN, DFNA5, and TPD52L1) and one writer (DNMT3A) (**Figures 5A, B**). The m5Cscore of each GC patient and prognostic information is summarized in **Table S7**. The alluvial diagram shows the attribute changes of individual GC patients (**Figure 5C**). Log-rank results depict that the OS of patients with a low m^5^Cscore is remarkably higher than patients with a high m^5^Cscore under the cutoff value of 9.92 (**Figures 5D, E**). The area under the curve (AUC) is 0.766, quantified by the pROC package (**Figure 5F**). Univariate and multivariate analysis demonstrates that age, N stage, M stage, and m^5^Cscore are the independent factors of prognosis (**Figures 5G, H**). Meanwhile, we found that m^5^Cscores significantly differed in distinct ACRG molecular subtypes. Patients in the EMT subgroup showed the highest m^5^Cscore compared to the other molecular groups (**Figure 6A**). Additionally, patients in m^5^C cluster B showed the lowest m^5^Cscore compared to other clusters (**Figure 6B**). In **Figure S3G–I**, GC patients with a high m^5^Cscore show a significantly higher stromal score and a lower tumor purity score. The results added to the evidence that a low m^5^Cscore was significantly related to immune activation and a high m^5^Cscore was correlated with stromal activation. m^5^Cscore could be a better marker to estimate the m^5^C modification of individual GC patients. Notably, patients with a low m^5^Cscore and who received adjuvant chemotherapy showed significant treatment advantages (**Figure 6C**). The result also demonstrated that the prediction value of m^5^Cscore was not affected by chemotherapy, and a low m^5^Cscore showed obvious survival advantage, regardless of whether patients received chemotherapy or not (**Figure 6C**). Moreover, older patients, intestinal histological subtype, and early GC patients were notably related to a low m^5^Cscore, which demonstrated that these GC patients were characterized by the m^5^C cluster B and immune-inflamed phenotype, with a better prognosis (**Figure 6D**).

### Validation of m^5^C modification in TCGA and other datasets

Data from the TCGA-STAD cohort and GEO were used for external and internal validation to determine the role of m^5^C modification and the prognostic value of m^5^Cscore. m^5^Cscore was employed to evaluate the individual m^5^C modification of the single patients in the TCGA dataset, among which 267 patients have a low m^5^Cscore and 69 patients have a high m^5^Cscore. Combined with the prognosis information, we revealed that patients with a low m^5^Cscore had a better prognosis (**Figure 6E**). ROC curve analysis showed that the AUC was 0.898, which was even higher than that in the training cohort (**Figure 6F**). We also noticed that patients with a high m^5^Cscore and without chemotherapy experienced the worst prognosis, while patients with a low m^5^Cscore and chemotherapy showed a favorable prognosis (**Figure 6G**). As shown in **Figures 6H, I**, patients in the high-m^5^Cscore group exhibit less extensive tumor mutation burden than patients in the low-m^5^Cscore group, with alternation rates of 88.41% and 92.88%, respectively. TMB analysis demonstrated that a high m^5^Cscore was significantly related to lower TMB, and showed a notable negative correlation (**Figures 6H, I**). Furthermore, the mean TMB of patients with a high or low m5Cscore was 2.31 and 1.26 mutations per MB. The violin plot also demonstrated that the TMB of patients in the high-m5Cscore group was significantly higher than that of patients in the low-m5Cscore group, and the *p*-value was 0.012 (**Figure S3J**).

**Figure 7:**
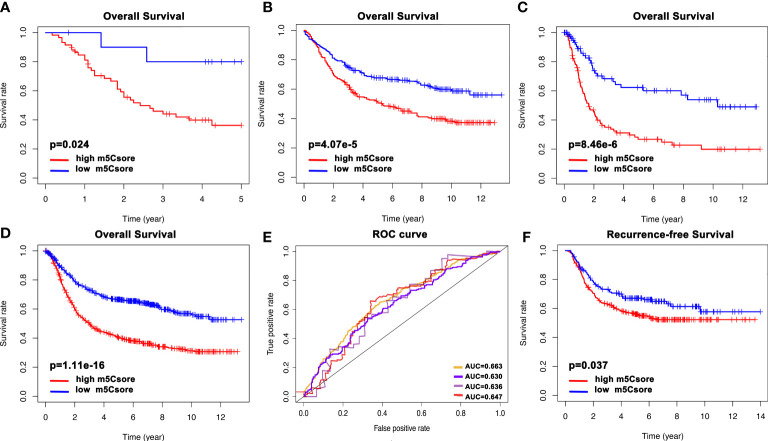
The validation of the m^5^Cscore system of individual GC patients from internal and external cohorts. **(A)** Overall survival analysis of m^5^Cscore in the GSE57303 cohort. **(B)** Overall survival analysis of m^5^Cscore in the GSE84437 cohort. **(C)** Overall survival analysis of m^5^Cscore in the GSE15459 cohort. **(D)** Overall survival analysis of m^5^Cscore in all five GEO cohorts. **(E)** The predictive value of m^5^Cscore in the four validation cohorts. The orange line represents all five GEO datasets with AUC 0.663; the purple line represents the GSE84437 cohort with AUC 0.630; the gray line represents the GSE57303 cohort with AUC 0.636; the red line represents the GSE15459 cohort with AUC 0.647. **(F)** Recurrence-free survival analysis of m^5^Cscore in the GSE26253 cohort.

Next, to further validate the stability of the m^5^Cscore system, the m^5^Cscore model was applied to other independent GC cohorts to confirm the prognostic value. **Figures 7A–C** show that GC patients with a low m^5^Cscore have a better prognosis in GSE57303, GSE84437, and GSE 15459. Moreover, we combined all the five GEO datasets together and found that the m^5^Cscore model was validated (**Figure 7D**). The ROC curve was drawn, and all AUCs were over 0.6 (**Figure 7E**). In addition, GSE26253, a new GEO dataset, was used to evaluate the predictive value of recurrence-free survival. **Figure 7F** confirms the ability of m^5^Cscore to predict RFS, which means the underlying potential mechanisms exist between m^5^C modification and tumor relapse to be elucidated.

## Discussion

Growing evidence revealed that aberrant RNA m^5^C methylation modification played a crucial role in tumorigenesis, progression, and patient prognosis by means of dynamic RNA epigenetic modification. In the current study, we analyzed that m^5^C regulators in GC explored the correlation between TME and m^5^C modification, as well as established an m^5^Cscore system to evaluate the prognosis of GC patients *via* the data from GEO datasets and the TCGA-STAD cohort. The m^5^Cscore model was further validated by internal and external datasets. These findings added clues for understanding the m^5^C modification of individual GC patients.

Sixteen m^5^C methylation regulators were involved in the analysis, including methyltransferases, demethylases, and RNA binding proteins. Although the exact number of m^5^C regulators and detailed mechanisms of m^5^C methylation are far from clear, the existing evidence has validated the essential function of m^5^C modification on different types of RNA, physiological, and pathological processes (7, 14). Among all the regulators, 13 regulators are significantly aberrantly expressed with 10 genes upregulated and 3 downregulated in GC samples. NSUN7 and DNMT2 are the only low-expression regulators out of the 11 methyltransferases. Sato et al. reported that NSUN7 was upregulated in low-grade glioma with an unknown mechanism (40). However, in GC, we suppose that the low expression of NSUN7 is caused by the loss of CNV frequency. Mei and colleagues found that NSUN2 was overexpressed in GC, which is consistent with our results, and they further validated that NSUN2 promotes GC cell proliferation *via* repressing p57(Kip2) in an m^5^C-dependent manner (41). In correlation analysis, we noticed that the methyltransferases tended to be related to each other, indicating the underlying interaction of mediating the m^5^C methylation modification. As for the readers, ALYREF and YBX1 were remarkably overexpressed in GC patients. Research on bladder cancer, breast cancer, HCC, and oral squamous cell carcinoma revealed that ALYREF and YBX1 were upregulated as well (22, 42–44). Intriguingly, high expression of ALYREF and YBX1 are also significantly correlated with the favorable prognosis of GC patients. All three erasers are notably related to the OS of GC patients despite the fact that only TET3 is significantly abnormally expressed in tumor samples.

Based on the expression of 16 m^5^C methylation regulators, three m^5^C modification patterns were distinguished. The three m^5^C modification clusters were characterized by different TME cell infiltration patterns. m^5^C cluster A was included in the immune-desert phenotype, characterized by immunosuppression; m^5^C cluster B belonged to the immune-inflamed phenotype, showing the activation of adaptive immunity; m^5^C cluster C was classified as immune-excluded phenotype, characterized by stroma and immunity activation. The GSVA analysis also revealed that m^5^C cluster B is enriched in cytokine–cytokine receptor interaction, natural killer cell-mediated cytotoxicity, antigen processing and presentation, Toll-like receptor signaling pathway, and chemokine signaling pathway. These results added to the evidence that the immune-inflamed phenotype, also known as a hot tumor, is characterized by immune cell infiltration and immune-related signal pathway stimulation in TME (45, 46). Additionally, we found that the immune checkpoints in m^5^C cluster B were notably overexpressed than the other two m^5^C clusters, which indicated that patients in m^5^C cluster B might benefit from immunotherapy. In the immune-excluded phenotype, TGF-β and EMT pathways are activated and abate the efficiency of immunotherapy (47). However, we observed the activation of TGF-β and EMT pathways in m^5^C cluster A, which was classified as the immune-desert phenotype. The anomaly may be due to the limited number of TGF-β and EMT pathway-related genes, which requires more data analysis and illustrates the complexity of m^5^C methylation modification. In survival analysis, m^5^C cluster B showed the most favorable prognosis, which is consistent with the above-mentioned immune-inflamed phenotype.

The m^5^Cscore system was established based on the expression of eight genes *via* the LASSO Cox regression method, namely, DNMT3A, RBPMS2, TNFRSF11A, NBEA, INHBB, RGN, DFNA5, and TPD52L1. Among all the genes calculated in the m^5^Cscore system, only DNMT3A is an m^5^C modification regulator; TNFRSF11A, INHBB, and DFNA5 are involved in TNF-related pathways (48–50); TPD52L1 participates in cell proliferation and calcium signaling; and RBPMS2, as an RNA binding protein, is involved in the regulation of cell differentiation and proliferation (51, 52). m^5^Cscore is a reliable marker to evaluate the prognosis of GC patients with an AUC of 0.766 in the ACRG training set and 0.898 in the TCGA validation set. m^5^Cscore was verified by other GEO datasets as well. Inevitably, m^5^Cscore is distinct in different m^5^C clusters, in which m^5^C cluster B had the lowest m^5^Cscore. We noticed that GC patients with the EMT molecular subtype show the highest m^5^Cscore, demonstrating poor prognosis. Furthermore, GC patients with a high m^5^Cscore tend to have a shorter RFS, indicating that m^5^C methylation may play an essential role in tumor recurrence.

## Conclusion

In summary, we revealed the potential regulatory mechanisms of m^5^C methylation modification on the GC TME. The characteristics of distinct m^5^C modification patterns might lead to the complexity and heterogeneity of individual GC TME. A far-reaching understanding of specific m^5^C modification patterns in GC will contribute to identifying TME cell infiltration and guiding clinical immunotherapy treatments.

## Data availability statement

The datasets presented in this study can be found in online repositories. The names of the repository/repositories and accession number(s) can be found in the article/**Supplementary Material**.

## Ethics statement

The study complied with the principles set forth in the Declaration of Helsinki. Access to the deidentified linked dataset was obtained from the TCGA and GEO databases in accordance with the database policy. For analyses of deidentified data from the TCGA and GEO databases, institutional review board approval and informed consent were not required.

## Author contributions

All authors searched the literature, designed the study, interpreted the findings, and revised the manuscript. QZ, JS, XS and KS carried out data management and statistical analysis and drafted the manuscript. JL, XG, KS and XQ helped with cohort identification and data management. QZ, JS, XS and KS performed project administration. All authors contributed to the article and approved the submitted version.

## Funding

This work was supported by grants from the National Natural Science Foundation of China (82003184) and the Health Science and Technology Project of Lianyungang (201914).

## Acknowledgments

The authors thank all individuals who participated in this study and donated samples.

## Conflict of interest

The authors declare that the research was conducted in the absence of any commercial or financial relationships that could be construed as a potential conflict of interest.

## Publisher’s note

All claims expressed in this article are solely those of the authors and do not necessarily represent those of their affiliated organizations, or those of the publisher, the editors and the reviewers. Any product that may be evaluated in this article, or claim that may be made by its manufacturer, is not guaranteed or endorsed by the publisher.
