# Supplementary material for: RNA m5C regulator-mediated modification patterns and the cross-talk between tumor microenvironment infiltration in gastric cancer
Source: Front Immunol. 2022 Oct 27;13:905057. doi: 10.3389/fimmu.2022.905057 (PMC9646743; doi:10.3389/fimmu.2022.905057)
Supplement: Supplementary file 2 [file DataSheet_1.pdf]

Materials and Method

GC dataset source and preprocessing

The flow chart of the current study was depicted in Fig S1A. Genomic data and clinical information from the Cancer Genome Atlas (TCGA) and the Gene-Expression Omnibus (GEO) were employed to analyze. Patients without follow up information were excluded. Eight eligible GC datasets (TCGA-STAD (Stomach Adenocarcinoma), GSE57303, GSE34942, GSE15459, GSE62254/ACRG, GSE62717, GSE26253 and GSE84437) were involved in further analysis. Sva package was used to correct the batch efforts of different GSE datasets via the "Combat" algorithm. Gene RNA sequencing data (FPKM value) were obtained from the GDC module of TCGA (Genomic Data Commons, https://portal.gdc.cancer.gov/) by the TCGAbiolinks package [32]. Detailed information of 7 cohorts was listed in Table S1. The somatic mutation information of GC patients was downloaded from TCGA. The date used in the analysis of Copy Number Variation (CNV) was from GSE62717, which belongs to the ACRG cohort as well. R (version 4.0.2) and Bioconductor packages were employed for data analysis.

Unsupervised clustering for 16 m^{5}C regulators

The genomic expression of 16 m^{5}C regulators was acquired from five integrated GEO cohorts to determine specific m^{5}C modification patterns. The 16 m^{5}C regulators were 11 methyltransferases (NSUN1, NSUN2, NSUN3, NSUN4, NSUN5, NSUN6, NSUN7, DNMT1, DNMT2, DNMT3A and DNMT3B), three demethylases (TET1, TET2 and

TET3) and two readers (ALYREF and YBX1). The different m^5C modification patterns were identified via an unsupervised clustering method. To maintain the stability of classification, the ConsensuClusterPlus package was employed to conduct the above steps and repeated 1000 times [33].

Gene set variation analysis (GSVA) and functional annotation

To explore the distinction on biological process among distinct m^5C modification patterns, GSVA enrichment analysis was performed via the GSVA packages. The gene sets of "c2.cp.kegg.v7.2.- symbols" were obtained from the MSigDB database for subsequent GSVA analysis. Adjusted P value less than 0.05 was defined as statistically significant.

Estimation of TME cell infiltration

The single-sample gene-set enrichment analysis (ssGSEA) quantified the abundance of specific cell infiltration. The gene set used to identify each kind of TME infiltrating immune cell was acquired from the research of Charoentong, which including different immune cells such as activated dendritic cell, natural killer T cell, regulatory T cell, activated CD8 T cell, macrophage and more (Table S2) [34]. Finally, the enrichment scores were employed to represent the relative abundance of specific TME infiltration cells for different GC samples.

Identification of differentially expressed genes (DEGs) between distinct m^5C phenotypes

The m^5C-related genes were identified under three different m^5C modification patterns. DEGs of distinct m^5C clusters was determined by the empirical Bayesian method [35]. The adjusted $P < 0.001$ was used as a criterion for identifying the DEGs.

Generation of m^5Cscore

To quantify the m^5C modification of the individual GC patient, a scoring system we termed as m^5Cscore was established. The steps to establish the m^5Cscore were as follows:

In the ACRG samples, the DEGs under distinct m^5Cclusters were firstly normalized, and the overlap genes were acquired. Subsequently, we conducted the prognostic analysis for the overlap genes via the univariate Cox regression method. The genes with significant prognosis and 16 m^5C regulators were incorporated in the LASSO multivariate Cox regression model via the R package "glmnet". The signature genes and coefficients in the m^5Cscore were generated based on the most applicable λ. The m^5Cscore formular we established was:

m^5Cscore= $\sum_{i=1}^{n} Coefi * Expi$

where *Coefi* is the coefficient and *Expi* is the normalized expression of each signature gene.

Statistical analysis

The correlations coefficients in the study were computed by distance correlation and Spearman analysis. One-way ANOVA and Kruskal-Wallis tests were employed to compare the difference of three or more groups. Univariate, multivariate, LASSO Cox regression and Kaplan- Meier analyses were conducted to construct and evaluate the risk signature via the “glmnet” and “survival” packages [36, 37]. The survivalROC package was employed to depict the ROC curve, which can predict the OS of GC patients. All of the statistical P were two sides, and less than 0.05 was considered statistically significant. R 4.0.2 software was employed to process the data in the research.

Figure S1. Study design and prognostic analysis of 16 m^5C regulators

(A) Overview of this research. (B) The mutation co-occurrence and exclusion analyses for 16 m^5C regulators. Co-occurrence, green; Exclusion, purple. (C) The prognostic analyses for 16 m^5C regulators in the five GC cohorts using a univariate Cox regression model. Hazard ratio >1 represented risk factors for survival and hazard ratio <1 represented protective factors for survival.

Figure S2. Unsupervised clustering of 16 m^5C regulators and immune cell infiltration in GC patients from five gastric cancer cohort.

(A-D) Consensus matrices of the five GC cohort for k = 2 - 5. (E) The component differences of immune cells among the three m^5C modification patterns analyzed by CIBERSORT. (F) The correlation of specific m^5C regulators and immune cells.

Figure S3. Unsupervised clustering of 16 m^5C regulators in the ACRG cohort and other analysis

(A-D) Consensus matrices of the ACRG cohort for k = 2 - 5. (E) The GO analysis of 245 DEGs in the three m^5Cclusters. (F) The KEGG signaling pathway enrichment of 245 DEGs in the three m^5Cclusters. (G) The stromal score of patients with low m^5Cscore and high m^5Cscore. (H) The immune score of patients with low m^5Cscore and high m^5Cscore. (I) The tumor purity score of patients with low m^5Cscore and high m^5Cscore. (J) The TMB of GC patients with low and high m5Cscore. NS, no significance; ***, p <0.001; *, p <0.05.

Figure S1

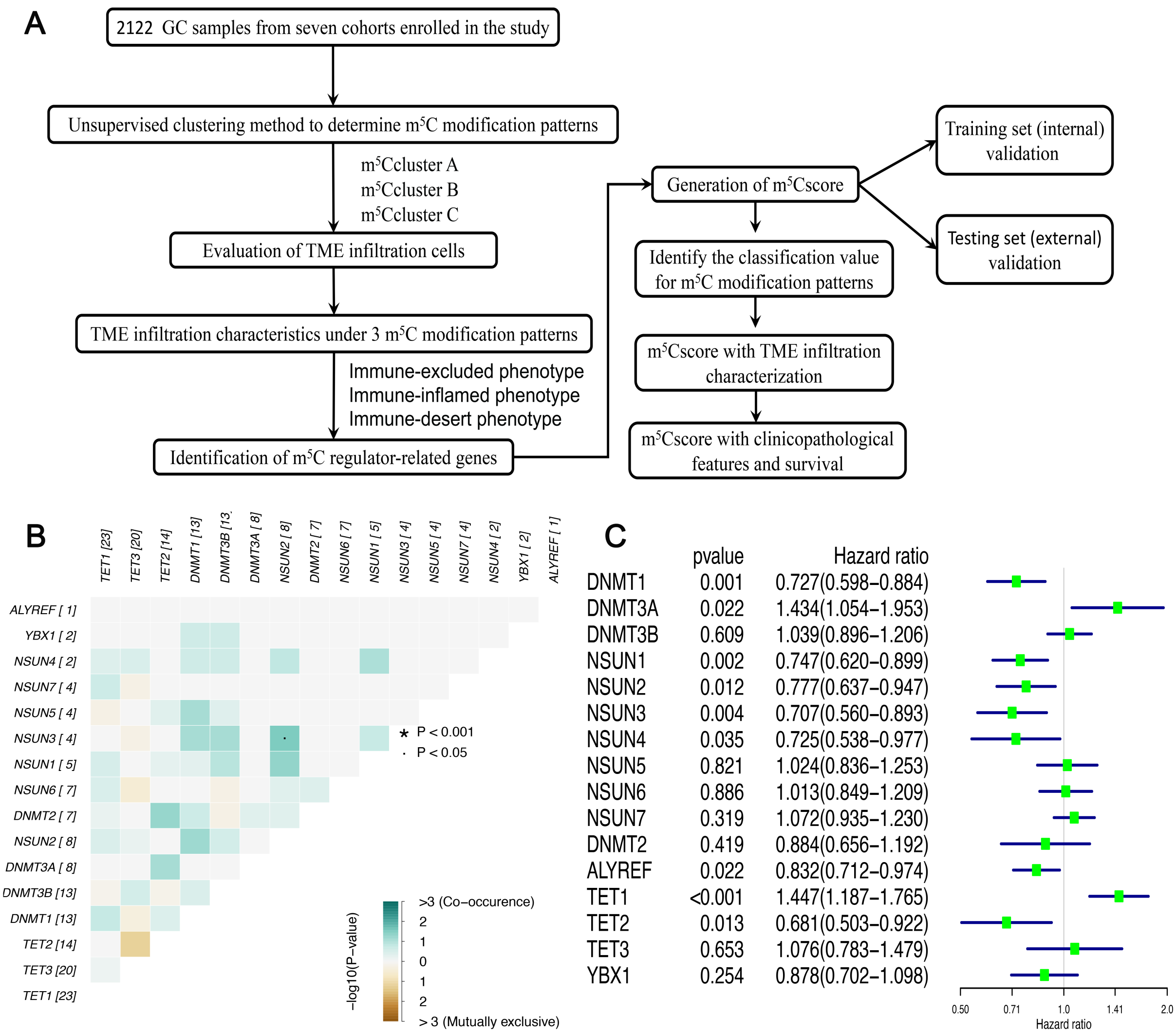

A
2122 GC samples from seven cohorts enrolled in the study
Unsupervised clustering method to determine m5C modification patterns
m5Ccluster A
m5Ccluster B
m5Ccluster C
Evaluation of TME infiltration cells
TME infiltration characteristics under 3 m5C modification patterns
Immune-excluded phenotype
Immune-inflamed phenotype
Immune-desert phenotype
Identification of m5C regulator-related genes
Generation of m5Cscore
Training set (internal) validation
Testing set (external) validation
Identify the classification value for m5C modification patterns
m5Cscore with TME infiltration characterization
m5Cscore with clinicopathological features and survival
B
TET1 [23]
TET3 [20]
TET2 [14]
DNMT1 [13]
DNMT3B [13]
DNMT3A [8]
NSUN2 [8]
DNMT2 [7]
NSUN6 [7]
NSUN1 [5]
NSUN3 [4]
NSUN5 [4]
NSUN7 [4]
NSUN4 [2]
YBX1 [2]
ALYREF [1]
★ P < 0.001
· P < 0.05
−log10(P−value)
>3 (Co−occurence)
2
1
0
1
2
> 3 (Mutually exclusive)
C
pvalue
Hazard ratio
DNMT1 0.001 0.727(0.598−0.884)
DNMT3A 0.022 1.434(1.054−1.953)
DNMT3B 0.609 1.039(0.896−1.206)
NSUN1 0.002 0.747(0.620−0.899)
NSUN2 0.012 0.777(0.637−0.947)
NSUN3 0.004 0.707(0.560−0.893)
NSUN4 0.035 0.725(0.538−0.977)
NSUN5 0.821 1.024(0.836−1.253)
NSUN6 0.886 1.013(0.849−1.209)
NSUN7 0.319 1.072(0.935−1.230)
DNMT2 0.419 0.884(0.656−1.192)
ALYREF 0.022 0.832(0.712−0.974)
TET1 <0.001 1.447(1.187−1.765)
TET2 0.013 0.681(0.503−0.922)
TET3 0.653 1.076(0.783−1.479)
YBX1 0.254 0.878(0.702−1.098)
0.50
0.71
1.0
1.41
2.0
Hazard ratio

Figure S2

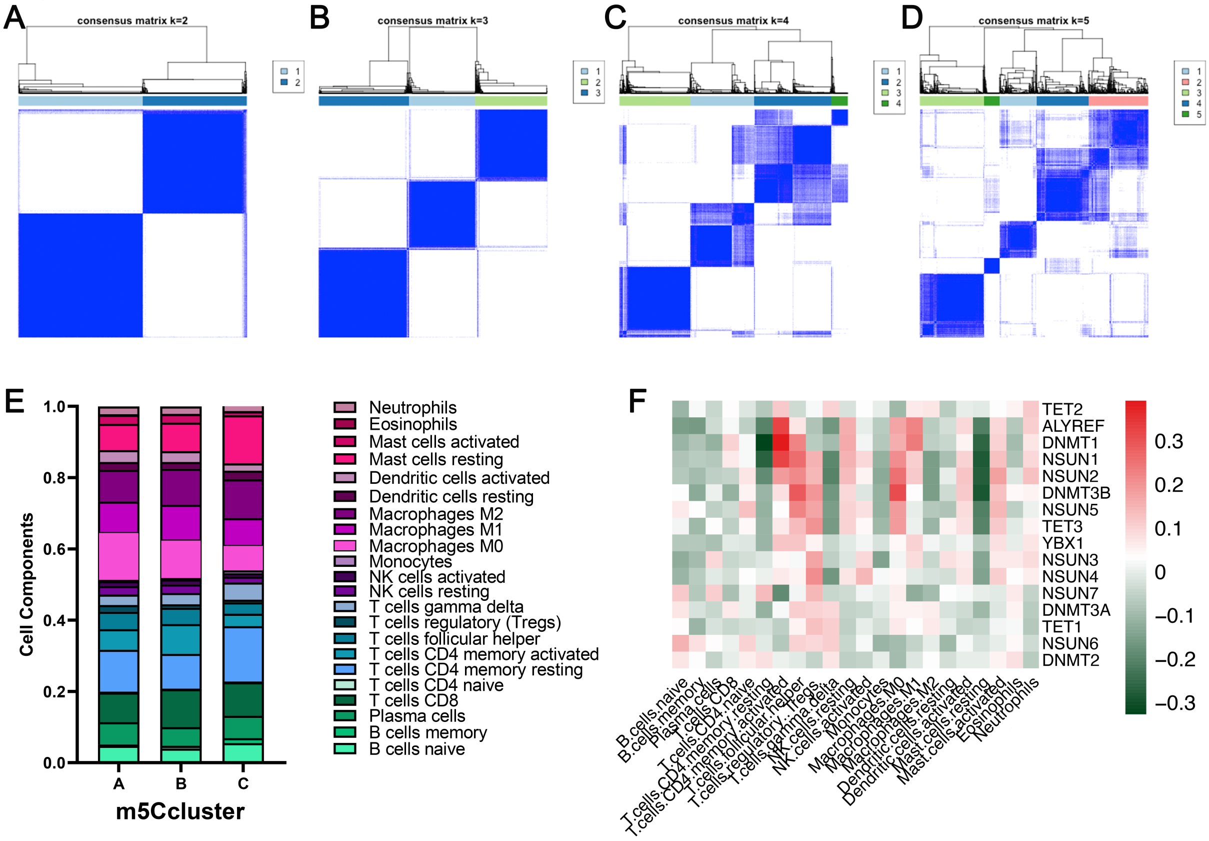

A
B
C
D
consensus matrix k=2
consensus matrix k=3
consensus matrix k=4
consensus matrix k=5
E
F
Cell Components
m5Ccluster
Neutrophils
Eosinophils
Mast cells activated
Mast cells resting
Dendritic cells activated
Dendritic cells resting
Macrophages M2
Macrophages M1
Macrophages M0
Monocytes
NK cells activated
NK cells resting
T cells gamma delta
T cells regulatory (Tregs)
T cells follicular helper
T cells CD4 memory activated
T cells CD4 memory resting
T cells CD4 naive
T cells CD8
Plasma cells
B cells memory
B cells naive
TET2
ALYREF
DNMT1
NSUN1
NSUN2
DNMT3B
NSUN5
TET3
YBX1
NSUN3
NSUN4
NSUN7
DNMT3A
TET1
NSUN6
DNMT2

Figure S3

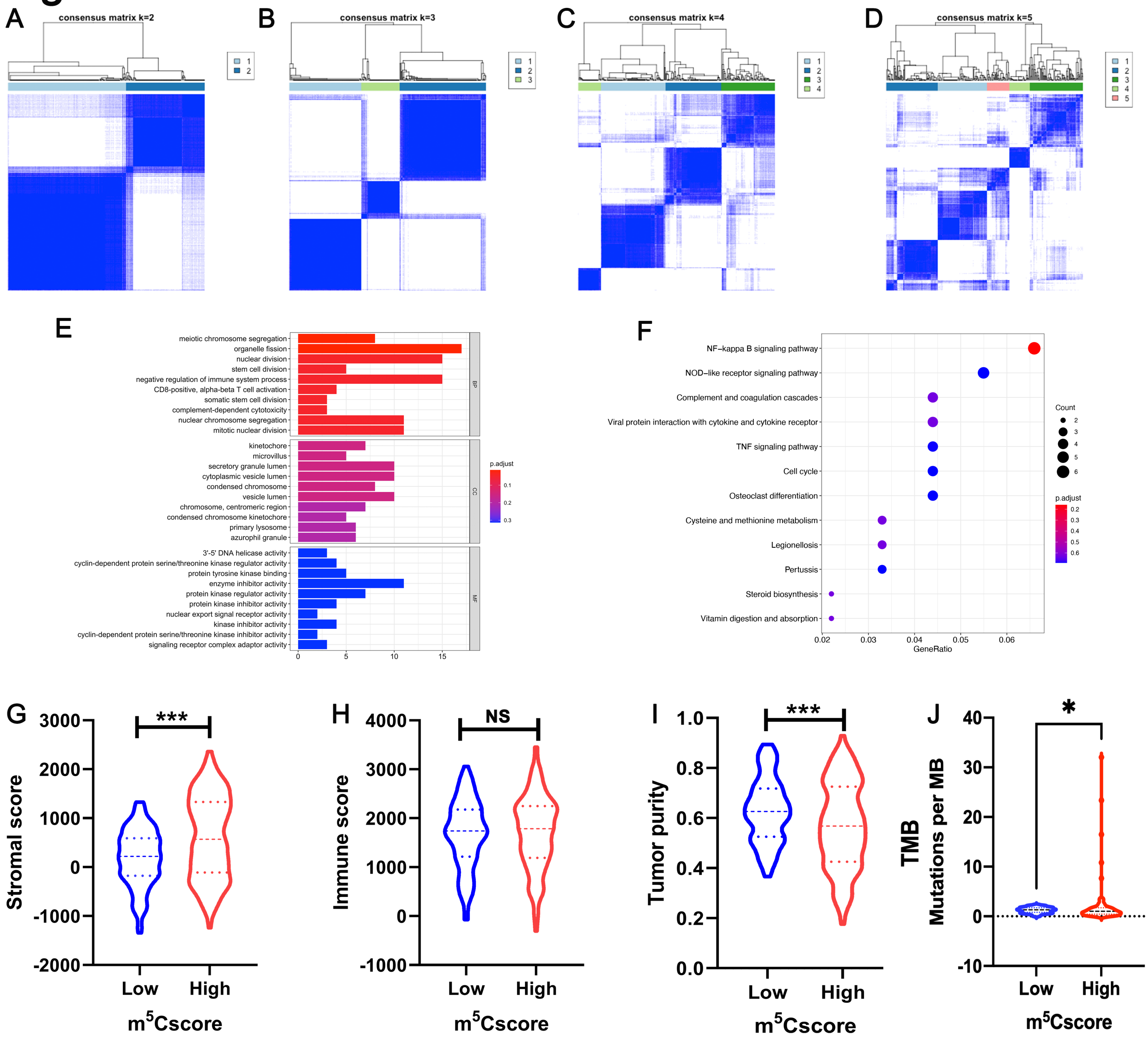

A
consensus matrix k=2
B
consensus matrix k=3
C
consensus matrix k=4
D
consensus matrix k=5
E
meiotic chromosome segregation
organelle fission
nuclear division
stem cell division
negative regulation of immune system process
CD8-positive, alpha-beta T cell activation
somatic stem cell division
complement-dependent cytotoxicity
nuclear chromosome segregation
mitotic nuclear division
kinetochore
microvillus
secretory granule lumen
cytoplasmic vesicle lumen
condensed chromosome
vesicle lumen
chromosome, centromeric region
condensed chromosome kinetochore
primary lysosome
azurophil granule
3'-5' DNA helicase activity
cyclin-dependent protein serine/threonine kinase regulator activity
protein tyrosine kinase binding
enzyme inhibitor activity
protein kinase regulator activity
protein kinase inhibitor activity
nuclear export signal receptor activity
kinase inhibitor activity
cyclin-dependent protein serine/threonine kinase inhibitor activity
signaling receptor complex adaptor activity
p.adjust
F
NF−kappa B signaling pathway
NOD−like receptor signaling pathway
Complement and coagulation cascades
Viral protein interaction with cytokine and cytokine receptor
TNF signaling pathway
Cell cycle
Osteoclast differentiation
Cysteine and methionine metabolism
Legionellosis
Pertussis
Steroid biosynthesis
Vitamin digestion and absorption
GeneRatio
Count
p.adjust
G
Stromal score
***
Low
High
m5Cscore
H
Immune score
NS
Low
High
m5Cscore
I
Tumor purity
***
Low
High
m5Cscore
J
TMB
Mutations per MB
*
Low
High
m5Cscore
